# Resistance to tumorigenesis in the african spiny mouse (*Acomys*) correlates with upregulation of multiple tumor suppressor genes

**DOI:** 10.1038/s41598-026-45001-6

**Published:** 2026-05-02

**Authors:** Marta Vitorino, Gonçalo G. Pinheiro, Inês Grenho, Ines M. Araujo, Bibiana I. Ferreira, Wolfgang Link, Gustavo Tiscornia

**Affiliations:** 1 Algarve Biomedical Center Research Institute-ABC-RI, Universidade do Algarve, Campus of Gambelas, Faro, 8005-139 Portugal; 2 Faculdade de Medicina e Ciências Biomédicas, Universidade do Algarve, Campus de Gambelas, Faro, 8005-139 Portugal; 3 Algarve Biomedical Center (ABC), Universidade Do Algarve, Campus de Gambelas, Faro, 8005-139 Portugal; 4 Sols-Morreale Biomedical Research Institute (IIBM), Spanish National Research Council (CSIC), Universidad Autónoma de Madrid (UAM), Madrid, Spain; 5 Molecular & Regenerative Medicine Lab, Centre of Marine Sciences (CCMAR/CIMAR LA), Universidade Do Algarve, Campus de Gambelas, Faro, 8005-139 Portugal

**Keywords:** Cancer, Zoology

## Abstract

Cancer remains a leading cause of morbidity and mortality worldwide, and the relationship between cancer and regeneration remains poorly understood. The spiny mouse (*Acomys* sp*.*) has attracted considerable attention due to its regenerative abilities. In this study, we compared the response of *Mus musculus (C57/BL6)* and *Acomys dimidiatus* mice to the DMBA/TPA papilloma inducing protocol. While both *Mus* and *Acomys* underwent DNA double strand breaks in their skin cells, mounted proliferative responses and underwent immune cell infiltration, only *Mus *developed tumors, whereas *Acomys* remained tumor-free. As an initial exploration of the molecular mechanisms underlying this resistance, we performed RNA sequencing on tissue samples from both species at baseline and at multiple time points during the carcinogenesis protocol. The data suggests distinctly different transcriptional responses between both species. *Acomys* showed significant upregulation of immune related genes, including a set of tumor suppressor genes, while *Mus* seemed to focus its response on modifying epidermis structure and regulation of the cell cycle.

**Supplementary Information:**

The online version contains supplementary material available at 10.1038/s41598-026-45001-6.

## Introduction

Intriguingly, tumorigenesis incidence seems to vary widely across the animal kingdom. While full pathological workups across appropriate age ranges are lacking, certain species in every taxon have been reported to have low rates of tumorigenesis. Species mentioned in the literature include bats, naked mole rats, elephants, and whales^1^. Possible underlying mechanisms hypothesized include lower somatic mutation rate, shorter telomeres, redundancy of tumor suppressors, a more efficient immune system, higher apoptosis rate and increased contact inhibition, among others^2^. The relationship between cancer, wound healing and regeneration is complex and poorly characterized. Upon injury, after an initial response to achieve hemostasis, organisms have two fundamental choices: regeneration or scarring. Regeneration involves recreation of the original tissue architecture, while scarring typically results in development of a scar tissue^3^. Regeneration involves a proliferative response that provides the cellular mass necessary for tissue reconstruction, which, if not strictly regulated, could lead to uncontrolled proliferation and development of tumors. A critical phase of wound healing is re-epithelization, where keratinocytes adjacent to the wound hyper-proliferate and migrate into the wound bed, both behaviors very similar to what occurs in cancer initiation and metastasis. In regeneration, proliferation is orderly, and importantly, responds to termination signals; if these signals are overridden, tumors may arise. Furthermore, wound healing and regeneration are related to cancer in that their major underlying molecular mechanism and pathways overlap^4–9^, to the point where it has been proposed that cancer is a wound that never heals^10,11^. The relationship between regeneration and cancer is reinforced by observations throughout the animal kingdom. One prevailing hypothesis suggests that high regenerative capacity tends to be associated with a low susceptibility to cancer, presumably due to trade-offs between both outcomes^12^. Planarians are capable of regeneration of entire organs even from small body fragments^13^, a process driven by pluripotent stem cells known as neoblasts^14^; however, the extent of this regenerative capacity varies across planarian species. Planarians from the genus *Schmidtea* are capable of full body regeneration, while those in the genus *Dugesia* exhibit more restricted regenerative abilities. Interestingly, *Dugesia* species also show a tendency to develop spontaneous tumors^15^ as well as after exposure to cadmium^16,17^, while *Schimidtea* does not, suggesting a link between reduced regenerative capacity and increased cancer susceptibility^18^. Knock-down of the planarian ortholog of p53 results cell cycle dysregulation and impaired regeneration^19,20^. Urodeles, including axolotls, salamanders, and newts, are capable of regenerating multiple tissues and organ systems. Despite axolotls having been observed to spontaneously develop skin tumors^21^, urodeles are thought to have low rates of tumorigenesis, and are resistant to cancer after carcinogen exposure^22^. Zebrafish can regenerate a range of tissues after injury^23^. While spontaneous tumor formation in zebrafish is rare^24,25^ exposure to carcinogens induces vigorous tumor formation in these animals. The power of the genetic toolbox available for this model has led to a number of studies linking cancer susceptibility to regenerative capability^26,27^.

The exceptional regenerative capacity of *Acomys sp*^28–34^ led us to establish this species as a research model several years ago. *Acomys* responds to wounding by mounting a regenerative response, in stark contrast with mammals generally, which repair their injuries by fibrotic scarring^34^. In response to full thickness 4 mm diameter ear pinna punch wounds (an injury comprising up to 35% of the ear pinna surface), *Acomys* mounts a strong proliferative response that re-establishes the original tissue architecture. In contrast, *Mus* simply heals the border of the wound by fibrotic scarring^28,34^. Dorsal skin wounds in *Acomys* also repair through a regenerative response, re-establishing dermis, epidermis, epidermal appendages, innervation and vascularization, as well as adipose and muscle layers^35^. Acute wound models of *Acomys* heart and kidney, while falling short of bona fide regeneration, show resistance to ischemic injury^36,37^. Injection of myotoxins into striated muscle, while initially causing severe cellular damage, are undetectable after 2 months^35^. None of these responses are observed in *Mus*. Perhaps most remarkably, a complete spinal cord transection in *Mus* leads to permanent loss of bladder control and hindlimb sensory/motor function but in contrast, *Acomys* with fully transected spinal cords regain bladder control within three weeks and recover up to 60% of motor function within 60 days post-injury^32^. These observations establish a powerful comparative framework in which two closely related species separated by only 23 million years of evolution show remarkably different responses to wounding (regeneration in *Acomys* vs. fibrotic scaring in *Mus*) in multiple tissues.

Interestingly, during wound healing studies in our *Acomys* colony we have never recorded uncontrolled proliferation or the development of spontaneous tumors in aged animals, despite their relatively long lifespan of six years. These observations suggest that regenerative capacity may be linked to specific mechanisms that regulate proliferation, potentially leading to cancer resistance as a secondary consequence. This observation prompted us to explore the susceptibility of *Acomys* to tumorigenesis.

In this study we used the well-established DMBA-TPA system for induction of skin tumors in mice^38,39^ to compare the tumor susceptibility of *Acomys dimidiatus* to that of *Mus*. This protocol involves initial topical treatment with a sub carcinogenic dose of 7,12-dimethylbenz[a]anthracene (DBMA), a chemical known to induce DNA double strand breaks. Cells (mainly keratinocytes of the basal epidermal layer and the hair bulge) that undergo an A → T transversion at the second nucleotide of codon 61 of H-ras acquire tumorigenic potential^40–42^, and when followed by repeated treatment with 12-O-tetradecanoylphorbol-13-acetate (TPA), a potent activator of PKC, a cascade of molecular signals cause sustained proliferative, inflammatory and survival signaling in the epidermis. These signals (MAPK/AP-1, NF-κB, COX-2/PGE₂, ROS, altered apoptosis thresholds, and activation of other pathways like Wnt) selectively expand and enable progression of the small population of initiated (mutant) cells created by DMBA^43^, eventually resulting in appearance of skin papillomas. Well established *Mus musculus* laboratory strains show varying degrees of tumorigenesis susceptibility. It has been reported that the distribution pattern for sensitivity to tumor promotion varies across *Mus* strains (SENCAR > DBA/2 ≥ CD-1 > C3H/He > > C57BL/6)^44^. Therefore, we chose C57BL/6, a strain reported to be relatively resistant to tumor induction (average papilloma burden of 2.5 papillomas per animal after 50 weeks) to determine whether *Acomys* would show even greater resistance to tumorigenesis when subjected to the same protocol. Our work suggests that *Acomys dimidiatus* is resistant to a DMBA/TPA papilloma inducing protocol that induces papilloma’s in C57/BL6 and provides insights into the underlying protective mechanisms operating in *Acomys.*

## Results

### Tumor induction resistance in *Acomys*

**Fig. 1:**
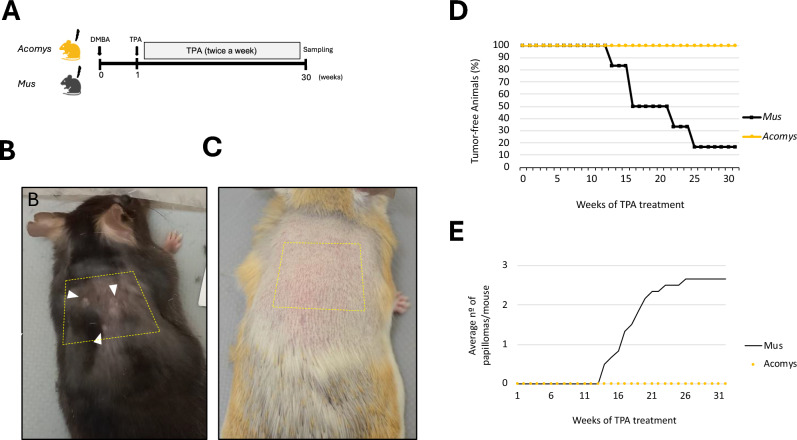
*Acomys dimidiatus* is resistant to tumor development induced by DMBA/TPA two stage protocol. (**A**) Schematic diagram for tumor induction by DMBA/TPA treatment on the dorsal skin of *M. musculus* and *A. dimidiatus*. (**B**) Dorsal view of *M. musculus* showing papillomas 30 weeks after DMBA/TPA treatment (**C**) Dorsal view of *A. dimidiatus* showing no papillomas 30 weeks after DMBA/TPA treatment. Yellow lines delimit treated regions. (**D**) Graphical representation of papilloma free animals vs. time of treatment and (**E**) average number of papillomas per animal over time of treatment.

Animals were treated with an initial dose of DMBA, followed by regular injections of TPA to induce proliferation, as described in Fig. 1A and Materials and Methods. Papillomas started appearing in *Mus* by week 13. After 30 weeks of the protocol, 5 out of 6 *Mus* (C57/Bl6) had developed papillomas in the treated area (Fig. 1B,D,E). The number of tumors in the *Mus* group after 30 weeks was 1, 1, 1, 3, 0 and 10, giving an average of 2,66 tumors/animal, in line with papilloma burden of C57/BL6 after DMBA-TPA treatment as reported in the literature^44^. In contrast, 6 out of 6 *Acomys* exhibited no sign of hyperplasia or tumor formation during the duration of the experiment (Fig. 1C–E). The difference in tumor number between species was statistically significant (Mann–Whitney test, p = 0.009). Figure 1E illustrates the average number of papillomas per animal over the course of the DMBA/TPA treatment. Clearly, the treatment that induces papillomas in C57/BL6 fails to induce papillomas in *A. dimidiatus*.

**Fig. 2:**
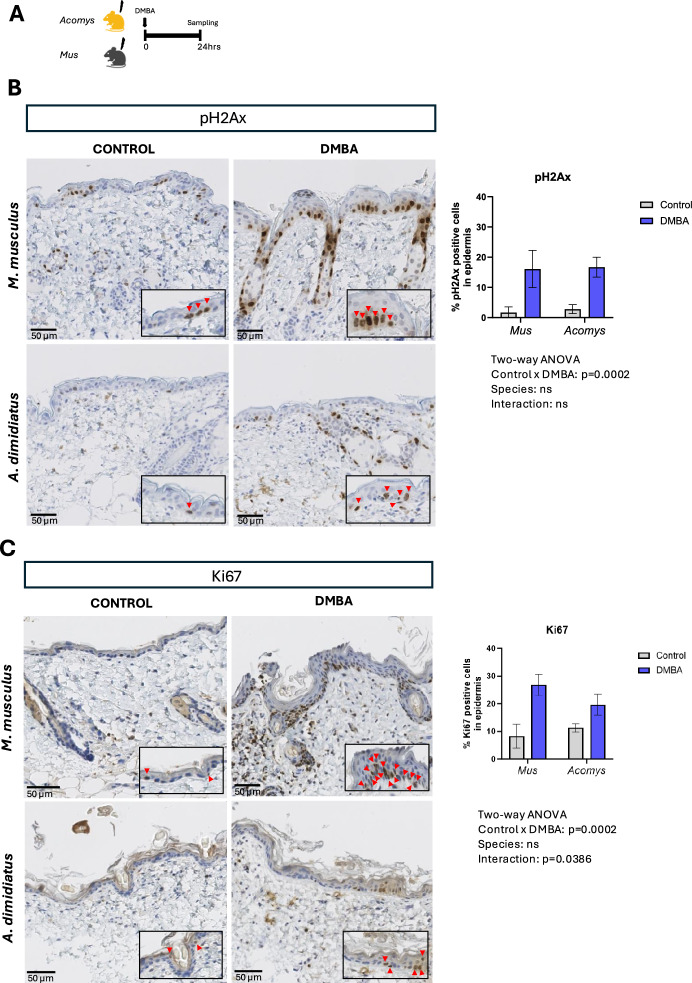
DMBA triggers double-strand-break formation in both *A. dimidiatus* and *M. musculus.* (**A**) Schematic illustration depicting the experimental approach to investigate short-term responses to DMBA treatment of both species. (**B**, **C**) immunohistochemistry staining and quantification of (**B**) pH2Ax-positive cells and (**C**) Ki67- positive cells in *M. musculus* and *A. dimidiatus* 24 hs after DMBA application. Scale bar: 50 μm.

First, we asked whether the initial DMBA treatment was inducing double strand breaks to similar levels in both species. Immunohistochemistry (IHC) for pH2Ax, a marker of DNA double strand breaks (DSB), 24 h after DMBA application (Fig. 2A) showed that DMBA treatment caused a marked increase in pH2Ax-positive cells in both *Mus* and *Acomys*, consistent with comparable genotoxic impact in both species (Fig. 2B) (for a more detailed statistical analysis, see Supplementary Data 5A).

Concurrently with presence of DSBs, we observed an early proliferative response in the epidermis of both species, as indicated by IHC against Ki67, a canonical proliferation marker. In both *Mus* and *Acomys*, DMBA increased Ki67-positive cells, but the magnitude of this proliferative response appeared greater in *Mus* than in *Acomys*, supporting a stronger DMBA-induced epidermal proliferation in *Mus* at this time point (Fig. 2C) (for a more detailed statistical analysis, see Supplementary Data 5B).

### Transcriptional analysis

We hypothesized that the difference in papilloma induction between both species could be attributed to early events occurring within the first month after treatment that effectively halt tumorigenesis in *Acomys*. To investigate this, we conducted a transcriptomic analysis at three different time points: 1 day (D1), 14 days (D14) and 28 days (D28) after the start of the protocol, comparing treated to non-treated animals (N = 4). PCA analysis revealed clustering of both *Mus* and *Acomys* samples, albeit with some heterogeneity, particularly in the *Acomys* group (Supplementary Fig. 1).

### Transcriptional profile at day 1 post- DMBA treatment

**Fig. 3:**
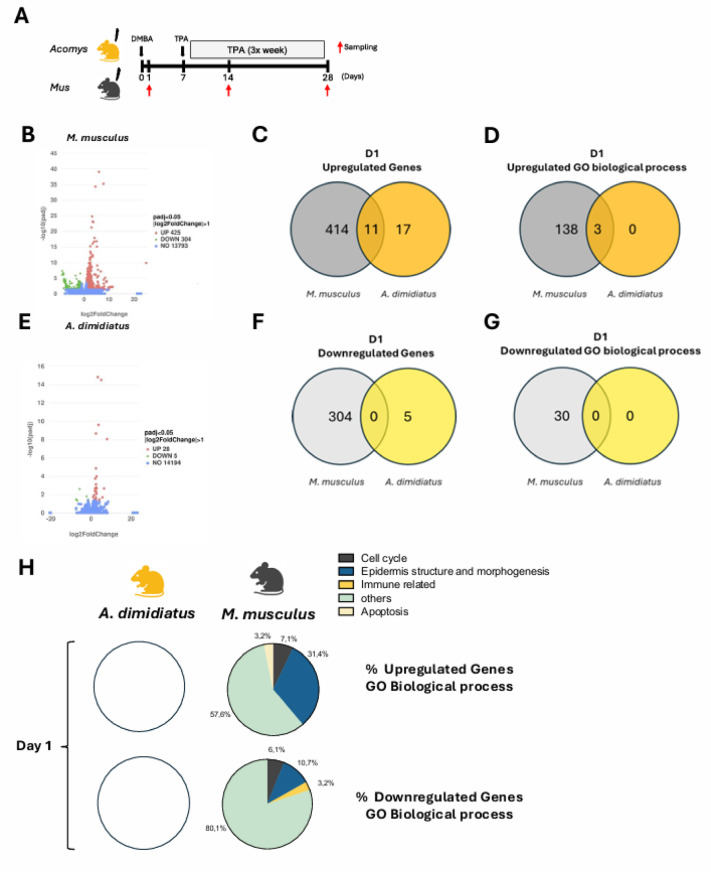
Transcriptomic profiles in *Mus* and *Acomys* 24 hs after DMBA administration. (**A**) Schematic illustration of transcriptomic experiment design. Red arrows indicate the times of sample collection. Volcano plots of genes differentially expressed in *M. musculus*. (**B**, **E**) Volcano plots of genes differentially expressed in *M. musculus* (**B**) and *A. dimidiatus* (**E**) one day after treatment with DMBA (D1). The log2 fold change (FC) indicates the mean expression level for each gene. Genes were scored as differentially expressed when log2 FC > 1, p < 0,05. Each dot represents one gene. Red dots represent upregulate genes, green dots represent downregulated genes and blue dots represent genes that are not differentially expressed. Venn Diagrams of upregulated genes (**C**) and GO biological processes (**D**), downregulated genes (**F**) and biological processes (**G**) in both species. (**H**) Percentage of upregulated and downregulated genes included in categories of GO biological processes. White circles are shown when there were no differentially expressed genes identified.

We queried differentially expressed genes (DEGs) (log2 = 1) and p-adj ≤ 0.05 (Fig. 3A). We comparatively examined genes exclusively upregulated in *Mus* but not in *Acomys* (414) and genes exclusively upregulated in *Acomys* but not in *Mus* (17) (Fig. 3B–E). Only 11 genes were upregulated in both species (Fig. 3C). A Panther analysis of upregulated *Mus* genes resulted in 138 enriched BP ontological categories, the genes of which could be grouped in the following biological themes: cell cycle and proliferation related processes (31.4%); processes related to epidermis structure and morphogenesis (7.1%); processes related to apoptosis (3.2%) and other processes (57.6%). In contrast, no BP ontological categories were significantly enriched for genes exclusively upregulated in *Acomys*, and only 3 BP ontological categories were enriched due to genes upregulated in both species (Fig. 3D,H).

We then examined genes exclusively downregulated in *Mus* but not *Acomys* (304) or exclusively downregulated in *Acomys* but not *Mus* (5) (Fig. 3B,E,F). No downregulated genes were shared between species (Fig. 3F). A Panther analysis of these downregulated *Mus* genes revealed 30 enriched BP ontological categories (Fig. 3G), the genes of which could be organized in the following biological themes: cell cycle and proliferation related processes (6.1%); processes related to epidermis structure and morphogenesis (10.7%); processes related to immunity (3.2%) and other processes (80.1%). In contrast, no BP ontological categories were significantly enriched for genes exclusively downregulated in *Acomys*, or genes downregulated in both species (Fig. 3H).

BP and KEGG pathway analysis suggested that response to the DMBA insult at 24 h did not result in either BP categories or KEGG pathway enrichment involving downregulated genes in either *Mus* or *Acomys.* However, the analysis did identify several BP categories and KEGG pathways significantly enriched for upregulated genes in both species (Supplementary Fig. 2). Therefore, the initial response to DMBA seems to be mediated by upregulation of specific gene sets in each species. We were particularly interested in the 17 genes upregulated (log2) in *Acomys* but not in *Mus*. Among this set we found several genes with intriguing functions related to tumorigenesis. *NQ01*, a NADP dehydrogenase (3.45-fold) is involved in detoxification and preventing formation of reactive oxygen species, possibly preventing cellular damage^45^. Two other genes with tumor suppressor functions exclusively upregulated in *Acomys* are *GNK1* (8.04-fold) and *SPINK7* (4.13-fold). Intriguingly, we found *CYP1A1*, a cytochrome P450 enzyme which is essential for the biotransformation of polycyclic aromatic hydrocarbons such as DMBA^46^ upregulated 5.1-fold in *Acomys*, but only 3.1-fold in *Mus*) (Table 1).

**Table 1:** List of genes upregulated in *Acomys* but not *Mus*, or upregulated in both species at D1 after treatment, with references linking them to relevant functions.

Upregulated in *Acomys* but not *Mus*	Log2 fold change	Function	Reference linking gene to function
*NQO1*	3.45	tumor suppressor	https://doi.org/10.5483/BMBRep.2015.48.11.190
*GKN1*	8.04	tumor suppressor	https://doi.org/10.5230/jgc.2014.14.3.147
*SPINK7*	4.13	tumor suppressor	https://doi.org/10.3390/ijms25115854

Upregulated in both *Acomys* and *Mus*	Log2 fold change *Mus/Acomys*	Function	Reference linking gene to function
*CYP1A1*	3.1/5.1	carcinogen inactivation	https://doi.org/10.1016/j.cotox.2020.07.001
*SLURP1*	2.36/2.59	tumor suppressor	https://doi.org/10.18632/oncotarget.24312
*KLK13*	2.84/2.73	tumor suppressor	https://doi.org/10.18632/oncotarget.2125
*KLK6*	6.04/2.13	tumor suppressor	https://doi.org/10.1186/s12943-015–0381-6
*KLK5*	2.34/1.92	tumor suppressor	https://doi.org/10.21873/anticancres.14219

### Transcriptional profile at day 14 post- DMBA-TPA treatment

**Fig. 4:**
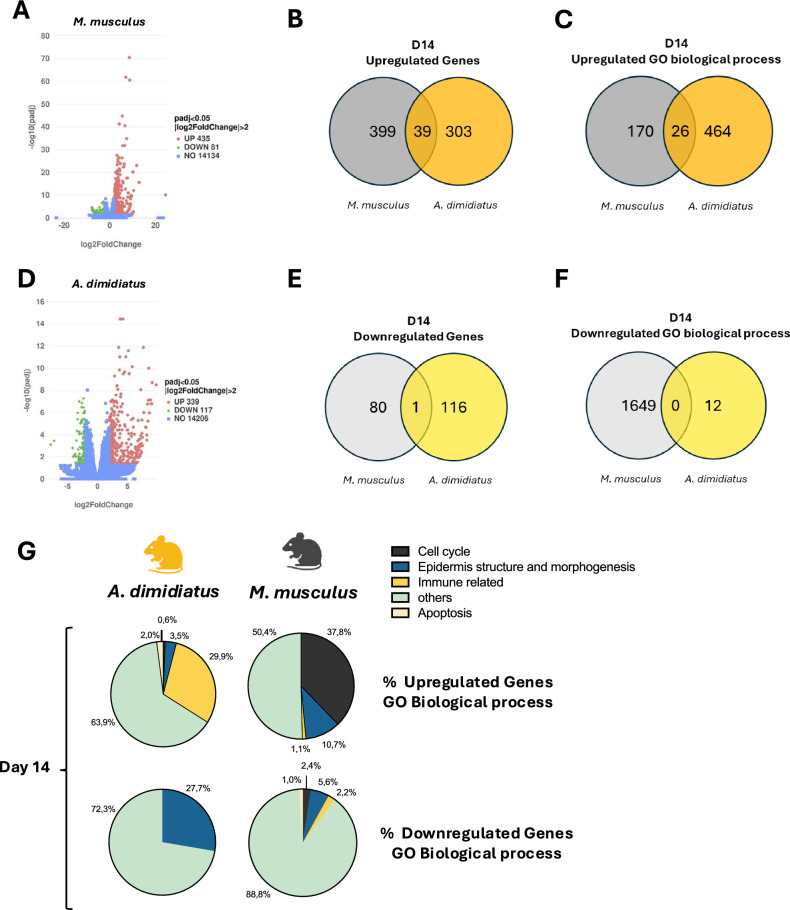
DMBA/TPA treatment induces activation of immune related genes in *A. dimidiatus* versus cell cycle regulation in *M. musculus*. (**A**) and *A. dimidiatus* (**D**) 14 days after treatment with DMBA (D14). The log2 fold change (FC) indicates the mean expression level for each gene. Genes were scored as differentially expressed when log2 FC > 2, p < 0,05. Each dot represents one gene. Red dots represent upregulate genes, green dots represent downregulated genes, and blue dots represent genes that are not differentially expressed. Venn Diagrams of upregulated genes (**B**) and GO biological processes (**C**), downregulated genes (**E**) and biological processes (**F**) in both species. (**G**) Percentage of upregulated and downregulated genes included in categories of GO biological processes.

*Mus* and *Acomys* samples at D14 (14 days after treatment with DMBA followed by TPA treatment at D7, D9 and D11) were compared to untreated samples harvested at D0 (N = 4). We queried DEGs (log2 ≥ 2) and p-adj ≤ 0.05 and comparatively examined genes exclusively upregulated in *Mus* but not in *Acomys* (399) and genes exclusively upregulated in *Acomys* but not in *Mus* (303) (Fig. 4A,B,D). Only 39 genes were upregulated in both species (Fig. 4B). To understand what BPs were associated with the set of genes upregulated exclusively in each species, we performed a Panther analysis revealed that genes upregulated exclusively in *Mus* resulted in 170 enriched BP ontological categories, while we found 464 BP ontological categories significantly enriched for genes exclusively upregulated in *Acomys*, and only 26 BP ontological categories enriched based on the 39 genes upregulated in both species (Fig. 4C). Analysis of the distribution of genes related to the enriched BP categories revealed striking differences in how each species responded to the treatment at this timepoint. In *Mus*, the number of genes in enriched BP categories could be grouped in the following biological themes: cell cycle and proliferation related processes (37.8%); processes related to epidermis structure and morphogenesis (10.7%); processes related to apoptosis (1.1%) and other processes (50.4%). In contrast, BP categories enriched in *Acomys* were related to immune response (29.9%), apoptosis (2%), cell cycle (0.6%), processes related to epidermic structure and morphogenesis (3.5%) and other processes (63.9%; Fig. 4C,G). We examined the identity and known functions of genes upregulated exclusively in *Acomys* vs. exclusively in *Mus*. Interestingly, we found that *Acomys* upregulated a total of 29 genes related to tumor suppression, with an average log2 fold increase of 4.31. Among these, several were upregulated to levels higher than log2 fold change = 6: *AHRR* (6.46); *CD80* (7.4); *CLCA2* (6.98); *GKN1* (6.78): *IL25* (8.41); *SHISA3* (6.37); *OAS3* (8.84) and *ST18* (6.37). In contrast, we found that *Mus* upregulated only 10 genes related to tumor suppression, and to an average log2 fold level of 3.04 (Table 2). To validate these observations, we performed an independent experiment, picked 4 genes showing positive differential expression in *Acomys* with relatively high adjusted count numbers in the initial experiment (*STAT1*, *IRF7*, *ISG15* and *GS02*) and quantitated relative expression in *Mus* vs *Acomys* by qPCR, finding expression levels in line with our initial sequencing results (Fig. 7).

**Table 2:** List of genes exclusively upregulated in *Acomys* but not *Mus* related to tumor suppressor functions; List of genes exclusively upregulated in *Mus* but not *Acomys*, related to tumor suppressor functions.

Genes exclusively upregulated in *Acomys* but not *Mus* at D14 post-treatment			
GENE	Deg in *Mus*	Deg in *Acomys*	Reference linking gene to tumor suppression
*AHRR*	NO	6.46	https://doi.org/10.3390/biology12040526
*AIM2*	NO	3.56	https://doi.org/10.1002/iids3.443
*BATF2*	NO	2.44	https://doi.org/10.3390/cancers13061263
*BCL2L15*	NO	2.35	https://doi.org/10.1038/s41419-019-1407-6
*CD80*	NO	7.4	https://doi.org/10.1080/2162402X.2021.1907912
*CLCA2*	NO	6.98	https://doi.org/10.1186/s13046-018-0692-8
*CRMP1*	NO	6.41	https://doi.org/10.1158/1535-7163.MCT-08-0091
*DHX58*	NO	2.64	https://doi.org/10.1158/0008–5472
*EIF2AK2*	NO	2.06	https://doi.org/10.3892/mmr.2017.7578
*G0S2*	NO	2.55	https://doi.org/10.1158/0008-5472.CAN-15-2265
*GBP2*	NO	4.71	https://doi.org/10.1093/carcin/bgs310
*GBP3*	NO	3.88	https://doi.org/10.3934/mbe.2023418
*GBP5*	NO	4.2	https://doi.org/10.7150/jca.94616
*GKN1*	NO	6.78	https://doi.org/10.1007/s10120-015–0483-2
*GLIPR1*	NO	2.62	https://doi.org/10.1186/s12943-016–0508-4
*IFI47*	NO	2.91	https://doi.org/10.1016/j.envpol.2022.120943
*IL24*	NO	8.41	https://doi.org/10.18632/oncotarget.6047
*IRF7*	NO	3.29	https://doi.org/10.1038/s41388-018–0624-2
*IRF8*	NO	2.31	https://doi.org/10.18632/oncotarget.16511
*ISG15*	NO	4.29	https://doi.org/10.3892/ijmm.2016.2845
*ISG20*	NO	2.47	https://doi.org/10.18632/aging.102714
*OAS3*	NO	8.84	https://doi.org/10.3389/fcell.2022.815480
*PTPRQ*	NO	5.95	https://doi.org/10.2147/OTT.S218125
*PTPRR*	NO	3.99	https://doi.org/10.1074/jbc.RA119.010348
*SAMSAN1*	NO	2.68	https://doi.org/10.1016/j.neo.2014.07.002
*SHISA3*	NO	6.37	https://doi.org/10.1371/journal.pone.0236192
*SOCS3*	NO	2.4	https://doi.org/10.1002/ijc.24172
*ST18*	NO	6.37	https://doi.org/10.1038/sj.onc.1208131
*STAT1*	NO	2.59	https://doi.org/10.18632/oncotarget.21790
	AVE DEG	4.41	

Genes exclusively upregulated in *Mus* but not *Acomys* at D14 post-treatment			
Gene	Deg in Mus	Deg in *Acomys*	Reference linking gene to tumor suppression
*Defb1*	2.63	NO	https://doi.org/10.21147/j.issn.1000-9604.2024.2024.04.01
*Dusp5*	2.68	NO	https://www.ajcr.us/ISSN:2156-6976/ajcr0038232
*Ehf*	4.07	NO	https://doi.org/10.1084/jem.20180749
*Grhl3*	2.53	NO	https://doi.org/10.1016/j.ymthe.2021.03.016
*Klk10*	2.83	NO	https://doi.org/10.1038/srep17426
*Mxd1*	2.12	NO	https://doi.org/10.1038/s41419-022-05279-6
*Rassf10*	2.22	NO	https://doi.org/10.3892/or.2022.8291
*Serpinb13*	4.92	NO	https://doi.org/10.1016/j.bbrc.2011.06.107
*Slc5a8*	4.4	NO	https://doi.org/10.31083/j.fbl2901016
*Trim62*	2.0	NO	https://doi.org/10.1002/path.4385
AVE DEG	3.04

**Fig. 5:**
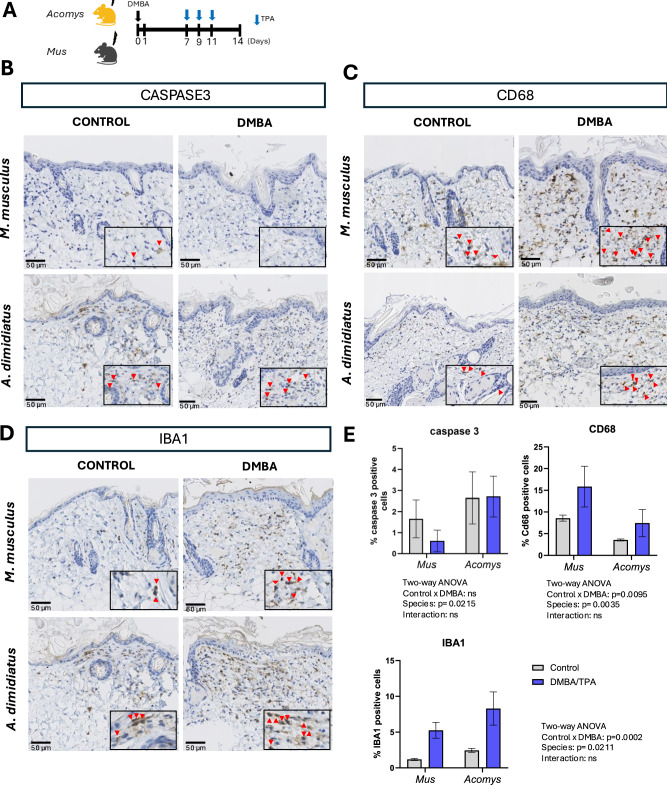
DMBA induces expression of CD68 in *M*. *musculus* and IBA1 in *A*. *dimidiatus*, but not cleaved-Caspase 3 in *A*. *dimidiatus* at D14. (**A**) Schematic illustration of the experimental approach to investigate short-term responses to DMBA treatment of both species. (**B**, **C**) immunohistochemistry staining of (**B**) cleaved-CASPASE3-positive cells, (**C**) CD68-positive cells and (**D**) IBA1-positive cells in *M. musculus* and *A. dimidiatus* at D14 after DMBA/TPA application. (**E**) Quantification of cleaved-caspase 3, CD68 and IBA1 positive cells in both *M. musculus* and *A. dimidiatus*. Scale bar: 50 μm.

Similarly, we examined genes exclusively downregulated in *Mus* but not in *Acomys* (80) and genes exclusively downregulated in *Acomys* but not in *Mus* (116) (Fig. 4A,D,E). Only 1 gene was downregulated in both species (Fig. 4E). When these gene sets were subjected to Panther analysis, a total of 1648 BP ontological categories were found to be enriched in *Mus*, with the associated genes organized into the following themes: processes related to epidermal structure and morphogenesis (5.6%), immune related processes (2.2%), cell cycle and proliferation related processes (5.6%), apoptosis-related processes (1%) and other processes (88.8%). Only 12 BP categories were enriched for *Acomys*, 8 of which were related to processes related to epidermal structure and morphogenesis (Fig. 4G).

In addition, a BP and KEGG pathway analysis indicated that the response to the DMBA/TPA treatment at D14 did not yield any enriched BP categories or KEGG pathway involving downregulated genes in either *Mus* or *Acomys.* However, the analysis revealed several BP categories and KEGG pathways significantly enriched for upregulated genes in both species (Supplementary Fig. 3). Collectively, the data from the D14 analysis suggest that the response in *Mus* primarily involves processes related to the cell cycle and morphological structures while *Acomys* regulates pathways associated with immune response, apoptosis, and tumor suppression.

Given the differential enrichment of immune related processes, particularly for *Acomys*, we conducted IHC against several infiltration markers (Fig. 5A). Although CD45, a pan-leukocyte marker, failed to work in *Acomys* (data not shown), both CD68 and IBA1, which are pan-markers for macrophages revealed different levels of infiltration in *Acomys* vs. *Mus* (Fig. 5C–E). For the macrophage marker CD68, a two-way ANOVA with species (*Acomys* vs *Mus)* and treatment (control vs DMBA) indicated that both baseline species differences and DMBA exposure are associated with substantial variation in macrophage infiltration, while statistical support for a difference in treatment effect between species is limited in this small-sample experiment (N = 3 animals per species × treatment group) (Fig. 5C,E). Biologically, *Mus* showed higher CD68-positive cell counts than *Acomys* at baseline and DMBA increased CD68 positivity in both species, with a pattern suggesting a larger response in *Mus* that should be interpreted cautiously given the non-significant interaction term and wide uncertainty (for detailed statistical analysis, see Supplementary Data 5C).

**Fig. 6:**
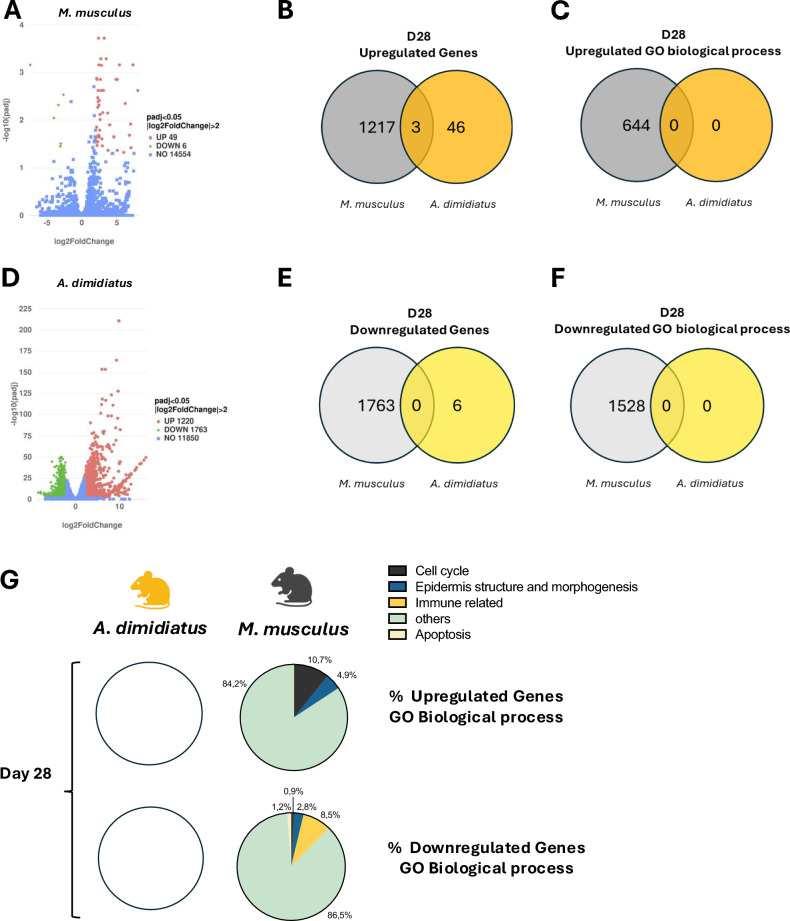
At day 28, *Mus* continues to show significant differential gene expression of genes, but *Acomys* return to baseline. (**A**, **D**) Volcano plots of genes differentially expressed in *M. musculus* (**A**) and A. dimidiatus (**D**) 28 days after treatment with DMBA (D1). The log2 fold change (FC) indicates the mean expression level for each gene. Genes were scored as differentially expressed when log2 FC > 2, p < 0,05. Each dot represents one gene. Red dots represent upregulate genes, green dots represent downregulated genes, and blue dots represent genes that are not differentially expressed. Venn Diagrams of upregulated genes (**B**) and GO biological processes (**C**), downregulated genes (**E**) and biological processes (**F**) in both species. (**G**) Percentage of upregulated and downregulated genes included in categories of GO biological processes. White circles are shown when there were no differentially expressed genes identified.

For Iba1, statistical analysis indicated that DMBA robustly enhanced Iba1 staining in both species, with *Acomys* showing higher absolute post-treatment levels and a somewhat stronger apparent increase than *Mus* (Fig. 5D,E).A two-way ANOVA with species (*Acomys* vs *Mus*) and treatment (control vs DMBA) confirmed strong main effects of both species and treatment, while the species × treatment interaction did not reach statistical significance, suggesting that although *Acomys* tends to respond more strongly, evidence for differential DMBA responsiveness between species is limited (for detailed statistical analysis, see Suplementary Data 5D). While CD68 and Iba1 are generally accepted as macrophage markers, it is noteworthy that *Mus* exhibits a higher number of CD68 + cells, while *Acomys* shows higher numbers of IBA1 + cells. While we cannot rule out that the antibodies recognize their targets with different efficiency in both species, this discrepancy suggests that these markers do not identify the same set of cells in both species.

Furthermore, we did IHC against the apoptotic marker cleaved-Caspase 3 to test whether apoptosis levels might correlate with tumorigenesis resistance (Fig. 5B,E). Untreated vs treated animal comparisons in each species showed that in *Mus* DMBA/TPA treatment seemed to reduce cleaved-caspase-3 levels (1.65 in untreated vs 0.6 in DMBA/TPA treated animals), but did not reach statistical significance (Welch test, p = 0.17). A similar comparison in *Acomys* showed no difference in acitvated caspase levels (2.64 in untreated vs 2.71 in treated animals, Welch test, p = 0.94). Group comparisons showed higher epidermal Caspase-3 labeling in *Acomys* than in *Mus*. These findings prompt the question of whether species-dependent difference in apoptotic response might play a role in tumorigenesis resistance, although this remains speculative given the low N. (for a more detailed ANOVA statistical analysis, see Suplementary Data 5E).

### Transcriptional profile at D28 post- DMBA-TPA treatment

*Mus* and *Acomys* samples at D28 (after initial treatment with DMBA and TPA treatment three times a week from D7 to D28) were compared to untreated samples harvested at D0 (N = 4). We queried differentially expressed genes (log2 = 2) and p-adj ≤ 0.05. A total of 1217 genes were exclusively upregulated in *Mus*, while only 46 genes were exclusively upregulated in *Acomys.* Only 3 genes were upregulated in both species (Fig. 6A,B,D). Panther analysis revealed that genes upregulated in *Mus* were associated with 644 significantly enriched BP ontological categories significantly enriched, with these genes relating to BPs in the following proportions: processes related to epidermal structure and morphogenesis (4.9%), processes related to cell cycle and proliferation (10.7%), and other processes (84.2%; Fig. 6C,G). No categories were enriched for genes exclusively upregulated in *Acomys* or upregulated genes shared by both species (Fig. 6C). Conversely, our analysis of downregulated genes revealed a total of 1763, and 6 genes exclusively downregulated in *Mus* vs *Acomys, respectively* with no genes downregulated in common, and 1528 and 0 processes exclusively downregulated in *Mus* vs *Acomys,* respectively (again with no processes downregulated in common between both species; Fig. 6A,D,E). When the set of downregulated genes in *Mus* was analyzed using the Panther pipeline, a total of 1528 BP categories were significantly enriched. The genes associated with these BPs corresponded to biological themes in the following proportions: epidermal structure and morphogenesis (2.8%), processed related to cell cycle and proliferation (0.9%), immune related processes (8.5%), apoptosis-related processes (1.2%), and other processes (86.5%; Fig. 6F,G). In summary, at D28 after initiation of the treatment, *Mus* continues to show significant differential expression of genes, mostly related to cell cycle control and epidermal structure and morphogenesis, but remarkably, the strong differential expression response seen in *Acomys* at D14 (303 and 116 up and downregulated genes respectively) seems to have declined to 57 and 7 up and downregulated genes respectively, suggesting a return of gene expression almost to baseline levels in *Acomys*. Furthermore, a BP and KEGG pathway analysis shows that response to the DMBA/TPA insult at D28 does not result in either BP categories or KEGG pathway enrichment involving downregulated genes in either *Mus* or *Acomys* but did reveal several BP categories and KEGG pathways significantly enriched for upregulated genes in both species (Supplementary Fig. 4).

**Fig. 7:**
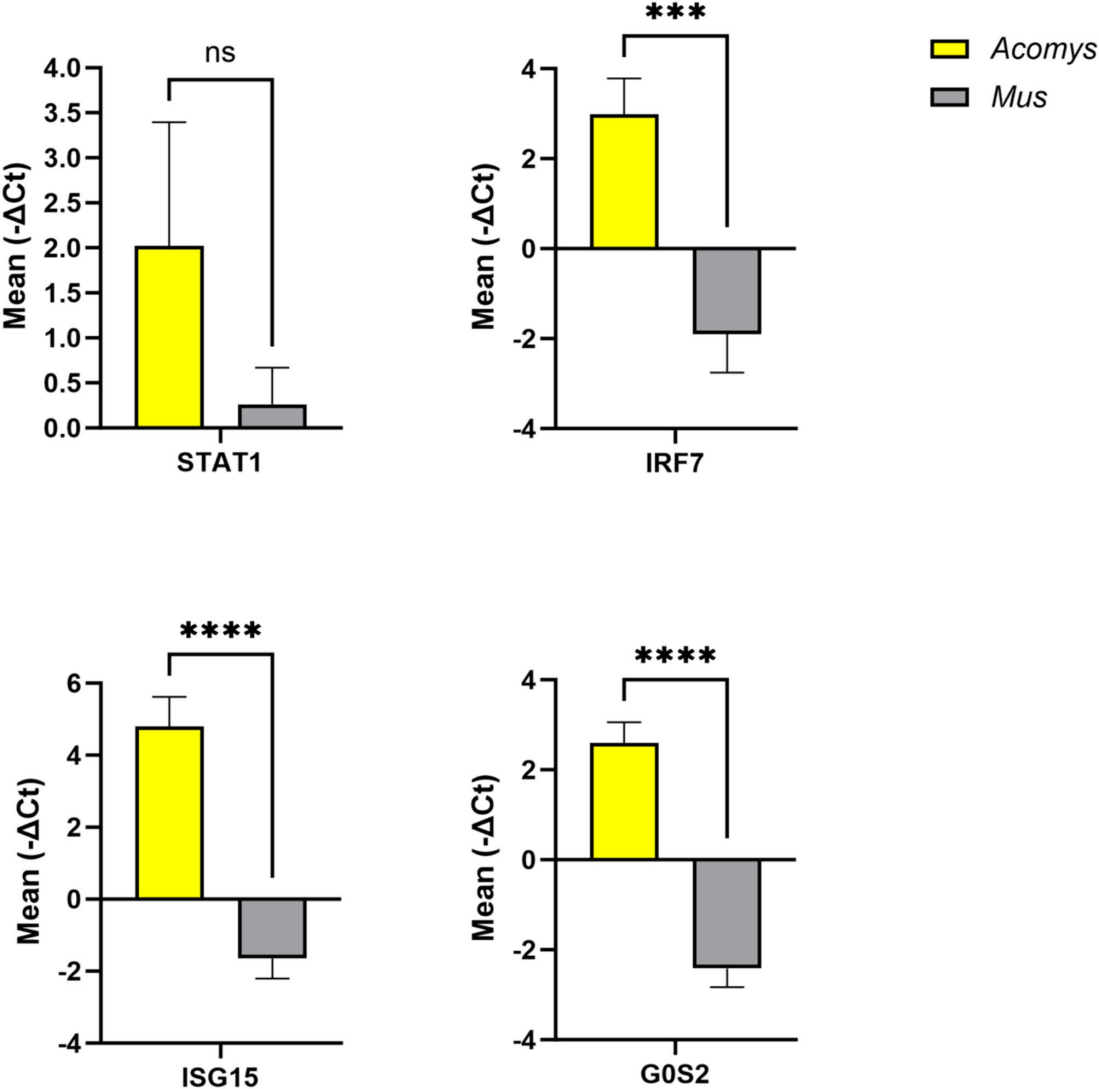
Differential induction of tumor suppressor-related genes in *Acomys* and *Mus* following DMBA/TPA treatment. Quantitative RT-PCR (qRT-PCR) analysis of Signal Transducer and Activator of Transcription 1 (*STAT1*), Interferon Regulatory Factor 7 (*IRF7*), Interferon-stimulated gene 15 (*ISG15*) and G0/G1 switch 2 (*G0S2*) at day 14 after treatment with DMBA/TPA compared to untreated skin in both animals, Acomys and Mus (n = 4). Glyceraldehyde-3-phosphate dehydrogenase (*GAPDH*) was used as reference gene. All qRT-PCR graphs are shown as the means ± SD. ns—non significant; ***P < 0.001; ****P < 0.0001; unpaired t test with Welch’s correction.

## Discussion

While *Acomys* is known for its remarkable regenerative properties^28–30,32–34^, to date the relationship between these traits and its incidence and vulnerability to cancer remains virtually unexamined. To investigate the sensitivity or resistance to tumorigenesis in *Acomys*, we employed a well-established tumor inducing protocol. We purposefully chose the C57/BL6 strain due to its relatively high level of resistance to papillomas induced by the DMBA/TPA protocol compared to other *Mus* strains^44,47,48^. The number of papillomas we obtained in C57/BL6 animals subjected to the DMBA/TPA protocol was in line with what has been published by multiple groups^39,47–53^. In the 6 *Mus* animals treated, the average number of papillomas after 30 weeks of treatment was 2.66 tumors/animal, while in contrast, we did not detect any tumors or signs of hyperplasia in the 6 *Acomys* animals treated. Therefore, for the levels of DMBA and TPA used in the tumor inducing protocol applied, there is a clearly statistically significant difference in the number of papillomas observed 30 weeks into the protocol, suggesting a degree of resistance of *Acomys*to tumorigenesis in the skin. Interestingly, a pre-print by White et al^54^, and a recently published report by Athar et al^55^, show similar observations.

We attempted to establish whether the initial genotoxic injury with DMBA was comparable in the 2 species by quantifying DSB and proliferation 24 h after treatment by IHC against pH2Ax and Ki67, respectively: we found that both species had statistically significant increases in the number of pH2Ax positive nuclei. It is clear that the lack of tumors observed in *Acomys* cannot be attributed to a lack of initial genotoxic injury in *Acomys*. Subsequent to DMBA treatment, TPA treatment was similar throughout the duration of the experiment, after which there was a clear difference in papilloma number. We did not test multiple groups with different concentrations of DMBA and TPA. It is possible that *Acomys* would indeed develop papillomas with higher dosages of DMBA and/or TPA, but regardless, our data clearly show a difference in tumor formation resistance between both species. We did not test whether H-ras mutations were present in the papillomas developed by *Mus*, as this has been repeatedly established in the literature. An attempt to detect H-ras mutations in *Acomys* at D14 was unsuccessful (data not shown), presumably due to high dilution of the signal, although we cannot rule out that for unexplained reasons H-ras mutations do not occur or do not become fixed in *Acomys*. The demonstration of whether DMBA causes H-ras mutations is pending and will likely involve a different experimental approach involving perhaps isolation and in vitro expansion of small numbers of in vivo treated cells.

Taken together, our data indicate that a) *Acomys* shows resistance to tumor induction using a protocol that clearly induces papillomas in *Mus*; 2) this difference is not due to failure of the treatment to induce DSBs in *Acomys*; 3) both species mount an initial proliferative response; 4) both species undergo immediate infiltration by immune cells. Our measurements of the apoptosis marker activated-caspase-3, while showing overall higher levels of apoptosis in *Acomys* vs *Mus* remain unconclusive (possibly due to low number of replicates (N = 3)); however, it is interesting to point out that Athar et al., 2025 found caspase-3–positive cells were significantly higher in DMBA/TPA-treated *Acomys* skin at week 30 compared with control animals.

We chose to explore the response of both species to the tumor inducing protocol during the first 28 days of treatment by transcriptomic analysis. The transcriptomic response of *Mus* and *Acomys* to the treatment differ significantly. At D1, *Mus* reacts with differential upregulation of gene expression in a total of 414 genes, while *Acomys* upregulates only 17 genes. The BP categories enriched in *Mus* focus mainly on cell cycle and epidermal structure and morphogenesis. In contrast, the 17 genes upregulated in *Acomys* include upregulation of the detoxification gene *NQ01*, as well as other genes with cancer prevention functions, including the tumor suppressor *SPINK 7*. The muted response in *Acomys* in terms of number of upregulated and downregulated genes at D1 was unexpected, and it could be argued that this indicates that the initial genotoxic insult was insufficient to trigger the tumorigenic process in *Acomys*. However, two observations counter this argument: first, the level of response to DMBA treatment at D1 in terms of amount of DSB and proliferation was similar in both species and second, the transcriptomic response, in terms of number of DEGs, is similar for both species (479 for *Mus*; 419 for *Acomys*) at D14 after treatment initiation. Interestingly, while the overall number of DEGs at D14 between the species was similar in magnitude, the DEGS were clearly distinct in terms of the functions those genes are involved in. The relatively low level of response at 24hs remains to be investigated. Our transcriptomic data suggests that by D14, the response of *Mus* to the genotoxic insult focuses on modifications to epidermal structure and an attempt to modulate proliferation and cell cycle related processes, while *Acomys*, in contrast, seems to unleash a multifaceted immune response. This suggests that the molecular strategies against tumorigenesis are radically different in these two species, and that *Acomys sp* may possess unique mechanisms to resist tumorigenesis, even under conditions that typically induce tumors in non-regenerative mammals. In particular, we found significant upregulation of genes that have been reported to be involved in tumor suppression in a number of contexts (Tables 1 and 2). This finding suggests the possibility that tumor suppression may play a role in *Acomys* tumorigenesis resistance. However, many tumor suppressor genes are known to be highly context dependent, with some TS acting in specific situations or even being drivers of tumorigenesis. The significance of this finding will need further confirmation.

It is important to recognize that the relationship between enhanced regenerative capabilities and tumor susceptibility is complex, and a deeper mechanistic understanding remains elusive both generally across animal groups and in *Acomys* particularly. While regenerative capacity may confer protection against tumorigenesis, rapid cell proliferation, an essential component of regeneration, can also increase the likelihood of mutations and tumor development. Interestingly, a recent report found significant roadblocks to reprogramming *Acomys* cells, suggesting that tumor suppressor pathways play an important role in *Acomys* regeneration and tumorigenesis resistance^56^.

Our results fit the pattern of animal model studies found in the literature associating high regenerative capabilities with tumorigenesis resistance, in this instance in a rodent phylogenetically close to humans. It is tempting to speculate that the key relation between regeneration and tumorigenesis resistance resides in the ability to control and cease proliferation during wound healing in an adaptive manner, and that these mechanisms are supercharged in a species that uses regeneration as a wound healing strategy. However, our transcriptomic analysis suggests that there are several layers to mechanisms deployed to surveil proliferation in *Acomys,* with the immune system playing a key role in controlling tumorigenic processes. This is evident from the observation that *Mus* seems to concentrate its control efforts on modulating the structure and state of the epidermis (keratinocyte proliferation and differentiation) and controlling cell cycle progression, while *Acomys* modulates 29.1% of the genes it upregulates at D14 of treatment in BP categories related to immune response^34,57–65^. In the context of the relationship between regeneration and tumorigenesis resistance, our results suggest a significant role of the *Acomys* immune system. While there is consensus in the literature regarding the significance of the *Acomys* immune system in regeneration and several studies have explored specific aspects^66,67^, much remains unknown. It has been speculated that regeneration in *Acomys* correlates with a blunted immune response involving a decrease in inflammatory cytokines^35^. In contrast, Gawriluk et al. observed that regeneration was associated with lower levels of pro-inflammatory cytokines (i.e., IL-6, CCL2, and CXCL1) and an increase in local levels of IL-12 and IL-17, correlating with an influx of T-cells into the wound area^67^. On D14, we did not find differential expressions of IL-6, CCL2, and CXCL1, but identified lower levels of other cytokines considered generally pro-inflammatory, such as IL-12, Il-17, IL-1a and IL-18 in *Acomys*. Also, macrophages have been shown to be required for epimorphic regeneration^66^. Our analysis in *Acomys* at D14 found that over 50% of BP categories related to a range of processes related to immunity, including T-cell proliferation, differentiation, migration, and cytokine production, NK chemotaxis and differentiation, macrophage associated processes and a number of cytokine-associated pathways. Our results suggest the immune system of *Acomys* is crucially positioned to determine the outcomes of wound healing, regeneration and cancer resistance. The general characterizations of immune responses in *Acomys* as simply blunt or heightened in comparison to that of non-regenerators seem to be premature and fail to capture the complexity and context-dependent response of the system. A thorough dissection of the *Acomys* immune response in relation to regeneration and tumor resistance is likely to keep researchers busy for a long time to come.

Our study, due to its exploratory nature, has limitations. While we clearly show differences in papilloma formation between *Mus* and *Acomys*, our transcriptomic data will need further corroboration. Considering that papillomas arise from small numbers of cells undergoing mutations in H-ras and the difficulty of detecting and selecting mutated areas of skin for analysis, the signal to noise ratio is low, suggesting our analysis uncovers major responses only. Future studies should increase the number of animals tested, test other tumor inducing protocols, make use of spatial omics approaches, utilize complementary in vitro approaches and include further controls, such as treatment with TPA only.

We have established a tractable and logistically convenient comparative model to specifically explore cancer resistance mechanisms in vivo in the laboratory. The *Acomys* model system may have significant implications for understanding the links between regeneration and tumorigenesis resistance and eventually, offer novel insights into potential therapeutic approaches to limit tumor development in humans.

## Methods

### Animals

Specimens of *A. dimidiatus* and *Mus musculus* C56BL6 were kept at the animal facility of the Algarve Biomedical Center Research Institute at the University of Algarve, using standard husbandry procedures for each species. *Acomys* were kept in a room with controlled temperature (26ºC), on a 13/11 h light/dark cycle and fed twice a week with mixed seeds supplemented with fresh fruits and vegetables, with water ad libitum, as described for this species^68^. All experiments were performed in accordance with European guidelines (2010/63/EU) for the care and use of laboratory animals, as well as Portuguese law (DL 113/2013). All experimental procedures were reviewed and approved by the Animal Welfare Body of the Universidade do Algarve and have been subjected for approval by the Direcção Geral de Alimentação e Veterinária of Portugal.

### DMBA/TPA treatment

To evaluate the induction of tumors by DMBA/TPA two stage protocol, the dorsal hair on a square of 2 × 2 cm on the back of *Mus musculus* C57BL/6N and *Acomys dimidiatus* animals (N = 6; 1:1 male/female ratio) was trimmed and the skin was treated topically with DMBA (Sigma-Aldrich; 100 μg in 200 μL acetone). *Mus* animals were between 7 and 9 week of age, while *Acomys* animals were approximately 3.5 months of age. The solution was applied evenly throughout the surface of the trimmed area. Starting one week after DMBA application, animals were treated twice weekly with an evenly applied solution of TPA (Sigma-Aldrich; 6.25 μg in 200 μL absolute ethanol) on the trimmed surface for 30 weeks. Tumor formation was assessed weekly. For a transcriptomic analysis of events during the first 28 days of treatment, *Mus* and *Acomys* animals were subjected to the same tumor inducing protocol and samples harvested as described above at day 1 (D1, 24 h after treatment with DMBA), day 14 (D14, after initial treatment with DMBA and TPA treatment on D7, D9 and D11) and day 28 (D28, after initial treatment with DMBA and TPA treatment 3 times a week from D7 to D28). Harvest involved humanely sacrificing the animals and collecting the entire 2 × 2 cm^2^ surface area treated with DMBA/TPA.

### Immunohistochemistry

Immunohistochemistry was performed in the Histopathology Core Facility at the Institute for Research in Biomedicine in Barcelona (Spain), following standard protocols. Briefly, *Acomys* dorsal skin was fixed in 4% paraformaldehyde overnight, embedded in paraffin, and cut into 3–5 μm sections. For immunostaining, the sections were dewaxed and epitope retrieval was performed with ER1 buffer (AR9961, Leica Biosystems) for Ki67 (Abcam, 15,580) for 30 min and with ER2 buffer (AR9640, Leica Biosystems) for Cleaved-Caspase 3 (Cell Signaling, 9661), CD45 (Cell Signaling, 98,819), H2AX (Cell Signaling, 9718) and CD68 (Byorbit, orb47985) for 20 min. Washings were performed using the BOND Wash Solution 10x (AR9590, Leica). Quenching of endogenous peroxidase was performed by 10 min of incubation with Peroxidase-Blocking Solution at RT (S2023, Dako, Agilent). Non-specific unions were blocked using 5% of goat normal serum (16,210,064, Life technology) mixed with 2.5% BSA diluted in wash buffer for 60 min at RT. Secondary antibody used was the BrightVision Poly-HRP-Anti Rabbit IgG Biotin-free, ready to use (DPVR-110HRP, Immunologic). Antigen–antibody complexes were reveled with the DAB (Polymer) (Leica, RE7230­CE). Sections were counterstained with hematoxylin (RE7107, Leica Biosystems) and mounted with Mounting Medium, Toluene-Free (CS705, Dako, Agilent) using a Dako CoverStainer. For Iba1 (019–19,741, Wako), samples were dewaxed and antigen retrieval process using citrate buffer pH6 for 20 min at 97ºC using a PT Link (Dako – Agilent) was performed. Blocking was performed with Peroxidase-Blocking Solution at RT (S2023, Agilent) and 5% of goat normal serum (16,210,064, Life technology) mixed with 2.5% BSA diluted in wash buffer for 10 and 60 min at RT. The secondary antibody used was the BrightVision poly HRP-Anti-Rabbit IgG, incubated for 45 min (DPVR-110HRP, ImmunoLogic). Antigen–antibody complexes were reveled with 3–3′-diaminobenzidine (K346811, Agilent). Sections were counterstained with hematoxylin (CS700, Dako, Agilent) and mounted with Mounting Medium, Toluene-Free (CS705, Agilent) using a Dako CoverStainer. Specificity of staining was confirmed staining with the rabbit IgG, polyclonal (NBP2-24,891, Novus bio-tec). Positive controls were as follows: brain sample for Iba1, intestine sample for Ki647, uterus for Caspase3, spleen for CD68 and testis for phospho-H2AX (*Mus*). Negative controls were samples treated with secondary antibody only and Rabbit IgG isotype control (Novus Biologicals, NBP2-24,891). Digital scanned brightfield images were acquired with a NanoZoomer-2.0 HT C9600 scanner (Hamamatsu, Photonics, France) equipped with a 20X objective and using NDP.scan2.5 software U10074-03 (Hamamatsu, Photonics, France). All images were visualized with the NDP.view 2 U123888-01 software (Hamamatsu, Photonics, France) with a gamma correction set at 1.8 in the image control panel of the NDP.view 2 U123888-01 software (Hamamatsu, Photonics, France). Immunohistochemical (IHC) number of positive cell determination was performed using QuPath software (version 0.5.0) on a total of 3 animals per experimental group. For each animal, one entire tissue section was analyzed using the automated Positive Cell Detection tool after defining the image type as Brightfield H-DAB25. This method allows for the detection and quantification of positively stained cells across the entire tissue sample. All statistical analyses were performed on animal-level data, using the mean value per animal obtained from 10 microscopic fields as the experimental unit (N = 3 animals per species × treatment group). Group differences were analyzed with a two-way analysis of variance (ANOVA) including species (*Mus* vs *Acomys*), treatment (control vs DMBA), and their interaction as fixed factors. For each marker, effect sizes were quantified using partial eta-squared, and 95% confidence intervals for group means were obtained by nonparametric bootstrap resampling of animals within each species × treatment combination. Assumptions of ANOVA (normality and homogeneity of variance) were checked using residual diagnostics. All analyses were conducted in R (R Foundation for Statistical Computing, Vienna, Austria) using standard linear model procedures and commonly applied guidelines for biological statistics and histopathology scoring.

### RNA sequencing

Total RNA was extracted from mice and *Acomys* skin tissues using Tryzol (Nzytech). Briefly, skin was first homogenized using Navy lysis kit (Next Advance) and extracted using Tryzol (Nzytech). RNA integrity was assessed using the Bioanalyzer 2100 system (Agilent Technologies). Messenger RNA was enriched from total RNA using poly-T oligo-attached magnetic beads. After fragmentation, the first strand cDNA was synthesized using random hexamer primers, followed by the second strand cDNA synthesis using dUTP. Libraries were subjected to end repair, A-tailing, adapter ligation, size selection, amplification, and purification. The libraries were checked with Qubit and real-time PCR for quantification and Bioanalyzer 2100 system (Agilent Technologies) for size distribution detection. After library quality control, different libraries were pooled based on the effective concentration and targeted data amount, then sequenced by Novogene Europe on the NovaSeq™ X Plus platform (Illumina).

Raw data (raw reads) of fastq format were processed through fastp software. In this step, clean data (clean reads) were obtained by removing reads containing adapters, reads containing poly-N and low-quality reads from raw sequence. At the same time, Q20, Q30 and GC content of the clean data were calculated. All the downstream analyses were based on high quality reads. The trimmed reads were mapped to either the *Mus musculus* (GCA_947599735.1) or *Acomys dimidiatus* (GCA_907164435.1) reference genomes using HISAT2 v2.05 software. We obtained over 20,000 reads for each species. Because the *Acomys* genome is incompletely annotated, we limited our downstream analysis to a subset of 16,542 genes which showed an unequivocal 1 to 1 ortholog relationship between both species, using *Mus* related databases to complete the analysis. The mapped reads of each sample were assembled by StringTie (v1.3.3b)^69^ in a reference-based approach. FeatureCounts v1.5.0-p3 was used to count the read numbers mapped to each gene and then FPKM of each gene was calculated based on the length of the gene and reads count mapped to this gene. Prior to differential gene expression analysis, for each sequencing library, read counts were adjusted using the edgeR R package (3.22.5) by scaling normalization factors to eliminate differences in sequencing depth between samples. Differential expression analysis for two conditions/groups was performed using the DESeq2 R package (1.20.0). The resulting p-value was adjusted using the Benjamini and Hochberg’s methods to control the error discovery rate. The corrected p-value ≤ 0.05 & log2 (foldchange)| was set at 1 for D1, and 2 for D14 and D28, as the threshold of significant differential expression. GO terms, Kegg pathways were analyzed using Panther (https://www.pantherdb.org) and NovoMagic (Novogene) bioinformatic software.

### Quantitative RT-PCR

For RNA analysis, tissues were treated with DMBA/TPA for fourteen days and collected in 750μL Trizol reagent. RNA was isolated using Trizol: Chloroform extraction (10:1) with isopropanol precipitation (1:1 isopropanol to Trizol) and two 70% EtOH washes before drying the pellet and resuspending the RNA in water. RNA quantity and quality was checked on a Nanodrop optical density reader. 2 µg of RNA was then converted to cDNA using SuperScript IV First-Strand Synthesis System cDNA synthesis Kit (ThermoFisher Scientific) according to the manufacturer instructions. RT-qPCR was performed using iTaq Universal SYBR Green Supermix in CFX384 Touch Real-Time PCR Detection System (BioRad) following the manufacturer protocol. Expression values (Mean -ΔCt) were calculated using *GAPDH* as the reference gene. All primers used are listed in Supplementary Table 1. Genes analyzed in quantitative RT-PCR were selected based on number of FKPM/counts obtained in the RNA sequencing experiment.

## Supplementary Information

Supplementary Information 1.   

Supplementary Information 2.

## Data Availability

Supplementary Information is available for this paper. Correspondence and requests for materials should be addressed to Gustavo Tiscornia.
