# Supplementary material for: Resistance to tumorigenesis in the african spiny mouse (Acomys) correlates with upregulation of multiple tumor suppressor genes
Source: Sci Rep. 2026 May 2;16:22459. doi: 10.1038/s41598-026-45001-6 (PMC13376787; doi:10.1038/s41598-026-45001-6)
Supplement: Supplementary file 1 — Supplementary Information 1. [file 41598_2026_45001_MOESM1_ESM.docx]

**Supplementary Figures, Legends, Tables and Data**

**Supplementary Table 1 -** Primers used for quantitative RT-PCR.

| ***Acomys*** | | |
| --- | --- | --- |
| Gene | Forward Primer | Reverse Primer |
| *GAPDH* | GGCATGGCCTTCCGTGTT | CAGTGGGCCCTCAGATGC |
| *STAT1* | CGACGACAGTTTTCCCATGG | AGAAGGTCGTGGAAACGGAT |
| *IRF7* | AGGCCGCTTTATGTTGCATC | CCTATTTTCCATGGCCAGGC |
| *ISG15* | GCTGAGACCAACAAAACCCC | GCATCATGGAGTTAGTCAGGG |
| *G0S2* | CAGCCCTTTCACAGCAGC | CCTGTGCCTTGTCTTTGTCC |
| ***Mus*** | | |
| Gene | Forward Primer | Reverse Primer |
| *GAPDH* | CCTTCCGTGTTCCTACCCCCAATGT | AGTGTAGCCCAAGATGCCCTTCAGT |
| *STAT1* | CGATGACAGTTTCCCCATGG | AGCTGTGAGAGGAGGTCATG |
| *IRF7* | ACAGGGCGTTTTATCTTGCG | CTGAGGCTCACTTCTTCCCT |
| *ISG15* | TCTGACTGTGAGAGCAAGCA | ACGGACACCAGGAAATCGTT |
| *G0S2* | AAGGAGATGATGGCGCAGAA | TGCACACCGTCTCAACTAGG |

**Supplementary Figure 1 - Principal component analysis (PCA) for *Acomys dimidiatus* and *Mus musculus* samples**. **(A)** PCA analysis of samples collected one day after application of DMBA in both *A. dimidiatus* and *M. musculus*. **(B)** PCA analysis of samples collected 14 days after application of short-term DMBA/TPA protocol in both *A. dimidiatus* and *M. musculus*. **(C)** PCA analysis of samples collected 28 days after application of short-term DMBA/TPA protocol in both *A. dimidiatus* and *M. musculus*. All PCA analyses included all samples (N=4) . MM_D0 represent untreated M. musculus samples , AC_D0 represent untreated *A. dimidiatus* samples , MM_D1_DMBA represent *M. musculus* samples collected 1 day after treatment with DMBA, AC_D1_DMBA represent *A. dimidiatus* samples collected 1 day after treatment with DMBA, MM_D14_DMBA represent *M. musculus* samples collected 14 days after treatment with DMBA/TPA, AC_D14_DMBA represent *A. dimidiatus* samples collected 14 days after treatment with DMBA/TPA, MM_D28_DMBA represent *M. musculus* samples collected 28 days after treatment with DMBA/TPA, AC_D28_DMBA represent *A. dimidiatus* samples collected 28 days after treatment with DMBA/TPA. PCA analyses was conducted using the Novomagic platform (Novogene Europe).

At 24hs, the PCA1 and PCA2 components explained 41.56% and 18.72% of variation in *Mus* and 41.19% and 22.41% in *Acomys,* respectively. At D14, the PCA1 and PCA2 components explained 53.02% and 19.73% of variation in *Mus* and 41.91% and 23.97% in *Acomys,* respectively. At 28D, PCA1 and PCA2 explain 77.4% and 11.64% of variability in *Mus*. *Acomys* samples showed one sample clustering with D0 samples, although overall, PCA1 and PCA2 explained 54.76% and 16.96% of the variance. . Overall, samples clustered as expected, although some variability was observed, particularly in *Acomys* samples. We presume that this may be due to our *Acomys* animals not being a purebred population. However, we have not determined the level of genetic variability of our *Acomys* colony and the low number of animals used in our experiments does not allow any analysis of distribution.

**Supplementary Figure 2 - DMBA treatment upregulated several GO biological processes and Kegg pathway. (A)** GO Biological processes upregulated and downregulated in *Mus musculus* and *Acomys dimidiatus*, 24h after the treatment with DMBA. **(B)** Kegg pathways upregulated and downregulated in *M. musculus* and *A dimidiatus*, 24h after the treatment with DMBA.

**Supplementary Figure 3 - DMBA treatment upregulated several GO biological processes and Kegg pathway. (A)** GO Biological processes upregulated and downregulated in *Mus musculus* and *Acomys dimidiatus*, 14 days after the treatment with DMBA. **(B)** Kegg pathways upregulated and downregulated in *M. musculus* and *A dimidiatus*, 14 days after the treatment with DMBA.

**Supplementary Figure 4 - DMBA treatment upregulated several GO biological processes and Kegg pathway. (A)** GO Biological processes upregulated and downregulated in *Mus musculus* and *Acomys dimidiatus*, 28 days after the treatment with DMBA. **(B)** Kegg pathways upregulated and downregulated in *M. musculus* and *A dimidiatus*, 28 days after the treatment with DMBA.

**Supplementary Data 5A:**

Immunohistochemical quantification using bootstrap group means (±95% CI) showed that in *Mus*, control pH2Ax values were relatively low (mean 1.64; 95% CI 0.08–3.76), whereas DMBA treatment produced high pH2Ax levels (mean 16.09; 95% CI 10.29–22.61); in *Acomys*, control skin also showed low pH2Ax levels (mean 2.79; 95% CI 1.62–4.46), while DMBA-treated *Acomys* exhibited substantially elevated pH2Ax (mean 16.68; 95% CI 13.68–20.19). A two-way ANOVA with species (*Acomys* vs *Mus*) and treatment (control vs DMBA) as fixed factors showed that DMBA markedly increased pH2Ax-positive cells compared with control (F(1,8)=43.85, p=0.00017, partial η²=0.85, indicating a very large effect), whereas the effects of species (F(1,8)=0.17, p=0.69, partial η²=0.02) and the species×treatment interaction (F(1,8)=0.02, p=0.90, partial η²=0.002) were negligible, supporting the conclusion that both species experienced similarly strong DSB induction after DMBA.

**Supplementary Data 5B:**

Bootstrap group means (±95% CI) in *Mus* showed control Ki67 was relatively low (mean 8.31; 95% CI 4.42–12.98), but DMBA produced a large increase (mean 26.79; 95% CI 22.37–29.32); for *Acomys*, control skin showed somewhat higher Ki67 labeling (mean 11.30; 95% CI 9.83–12.88) and a relatively lower increase after DMBA (mean 19.65; 95% CI 15.37–22.64) compared to *Mus*. A two-way ANOVA with species (*Acomys* vs *Mus*) and treatment (control vs DMBA) showed a strong main effect of treatment (F(1,8)=42.92, p=0.00018) and a substantial species×treatment interaction (F(1,8)=6.11, p=0.039), with a smaller, non-significant main effect of species (F(1,8)=1.02, p=0.34); partial eta-squared values were η²=0.84 for treatment, η²=0.11 for species, and η²=0.43 for the interaction, which, despite the small sample size and absence of confidence intervals on effect sizes, are consistent with a very large effect of DMBA and a large interaction effect on Ki67 levels.

**Supplementary Data 5C:**

More specifically, the two-way ANOVA showed large main effects of species and treatment, and a smaller, non-significant species×treatment interaction: species, F(1,8)=16.70, p=0.0035, partial η²=0.68; treatment, F(1,8)=11.54, p=0.0094, partial η²=0.59; species×treatment, F(1,8)=1.06, p=0.33, partial η²=0.12 . Bootstrap group means (±95% confidence intervals) were: in Acomys, CD68-positive cells were low in controls (mean 3.54; 95% CI 3.28–3.69) and increased after DMBA (mean 7.43; 95% CI 4.02–10.20); in Mus, control skin already showed higher CD68 (mean 8.57; 95% CI 8.00–9.35), and DMBA produced a further increase (mean 15.86; 95% CI 11.76–20.99) . These numerical results support the conclusion that Mus exhibits higher macrophage infiltration than Acomys at baseline and after DMBA, and that DMBA enhances CD68 positivity in both species, while any apparent species difference in the magnitude of the DMBA effect remains statistically unconfirmed in this dataset .

**Supplementary Data 5D:**

Bootstrap group means (±95% CI) were: in Mus, Iba1-positive cells were low at baseline (mean 1.18; 95% CI 1.07–1.36) but increased to intermediate levels after DMBA (mean 5.25; 95% CI 4.40–6.52), whereas in Acomys, control Iba1 levels were higher than for Mus (mean 2.45; 95% CI 2.18–2.74) and rose further following DMBA treatment (mean 8.29; 95% CI 6.29–10.85) . A two-way ANOVA with species and treatment as fixed factors revealed a strong main effect of treatment (F(1,8)=43.43, p=0.00017) and a significant main effect of species (F(1,8)=8.17, p=0.021), whereas the interaction term was non-significant (F(1,8)=1.39, p=0.27) . Partial eta-squared values were η²=0.84 for treatment and η²=0.51 for species, indicating very strong effects of DMBA exposure and substantial baseline/species differences in Iba1 levels, while the interaction η²=0.15 provides only modest statistical support for a difference in DMBA response between species .

**Supplementary Data 5E:**

Bootstrap group means (±95% CI) were: in *Mus*, caspase-3–positive cells were lower overall and showed a non-significant tendency to decrease with DMBA (control mean 1.65; 95% CI 0.61–2.23; DMBA mean 0.60; 95% CI 0.23–1.19), whereas in *Acomys*, cleaved caspase-3–positive cells were similar in control and DMBA-treated skin (control mean 2.64; 95% CI 1.50–3.95; DMBA mean 2.71; 95% CI 1.60–3.40). These data indicate higher epidermal caspase-3 labeling in *Acomys* than *Mus*, with no clear DMBA-induced increase in apoptosis in either species under the present sample size, and only a small, uncertain interaction pattern. A two-way ANOVA with species (*Acomys* vs *Mus*) and treatment (control vs DMBA) indicated that variation was dominated by species, with only small contributions from treatment and the species×treatment interaction: there was a significant main effect of species (F(1,8)=8.11, p=0.022), but non-significant effects of treatment (F(1,8)=0.82, p=0.39) and of the species×treatment interaction (F(1,8)=1.05, p=0.33) . Partial eta-squared values were 0.50 for species, 0.09 for treatment, and 0.12 for the interaction, suggesting a substantial overall difference between species (higher levels of activated caspase-3 in *Acomys* compared with *Mus*) but only modest and statistically uncertain effects of DMBA and its interaction with species in this small-sample design .
