# Supplementary material for: Resistance to tumorigenesis in the african spiny mouse (Acomys) correlates with upregulation of multiple tumor suppressor genes
Source: Sci Rep. 2026 May 2;16:22459. doi: 10.1038/s41598-026-45001-6 (PMC13376787; doi:10.1038/s41598-026-45001-6)
Supplement: Supplementary file 2 — Supplementary Information 2. [file 41598_2026_45001_MOESM2_ESM.pdf]

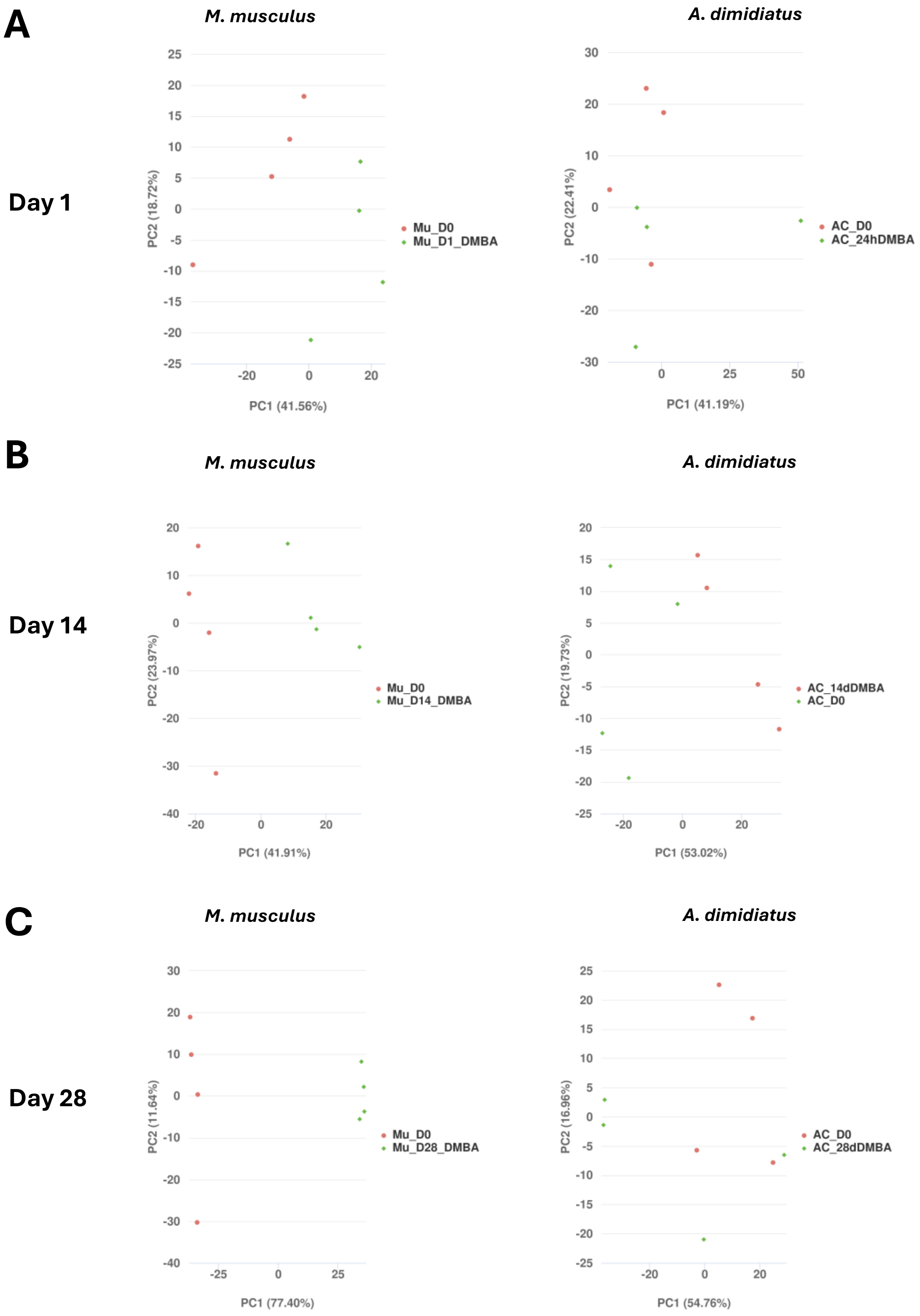

Supplementary Figure 1 - Principal component analysis (PCA) for *Acomys dimidiatus* and *Mus musculus* samples. **(A)** PCA analysis of samples collected one day after application of DMBA in both *A. dimidiatus* and *M. musculus*. **(B)** PCA analysis of samples collected 14 days after application of short-term DMBA/TPA protocol in both *A. dimidiatus* and *M. musculus*. **(C)** PCA analysis of samples collected 28 days after application of short-term DMBA/TPA protocol in both *A. dimidiatus* and *M. musculus*. MM_D0 represent M. musculus samples at day 0, AC_D0 represent *A. dimidiatus* samples at day 0, MM_D1_DMBA represent *M. musculus* samples collected 1 day after treatment with DMBA, AC_D1_DMBA represent *A. dimidiatus* samples collected 1 day after treatment with DMBA, MM_D14_DMBA represent *M. musculus* samples collected 14 days after treatment with DMBA/TPA, AC_D14_DMBA represent *A. dimidiatus* samples collected 14 days after treatment with DMBA/TPA, MM_D28_DMBA represent *M. musculus* samples collected 28 days after treatment with DMBA/TPA, AC_D28_DMBA represent *A. dimidiatus* samples collected 28 days after treatment with DMBA/TPA.

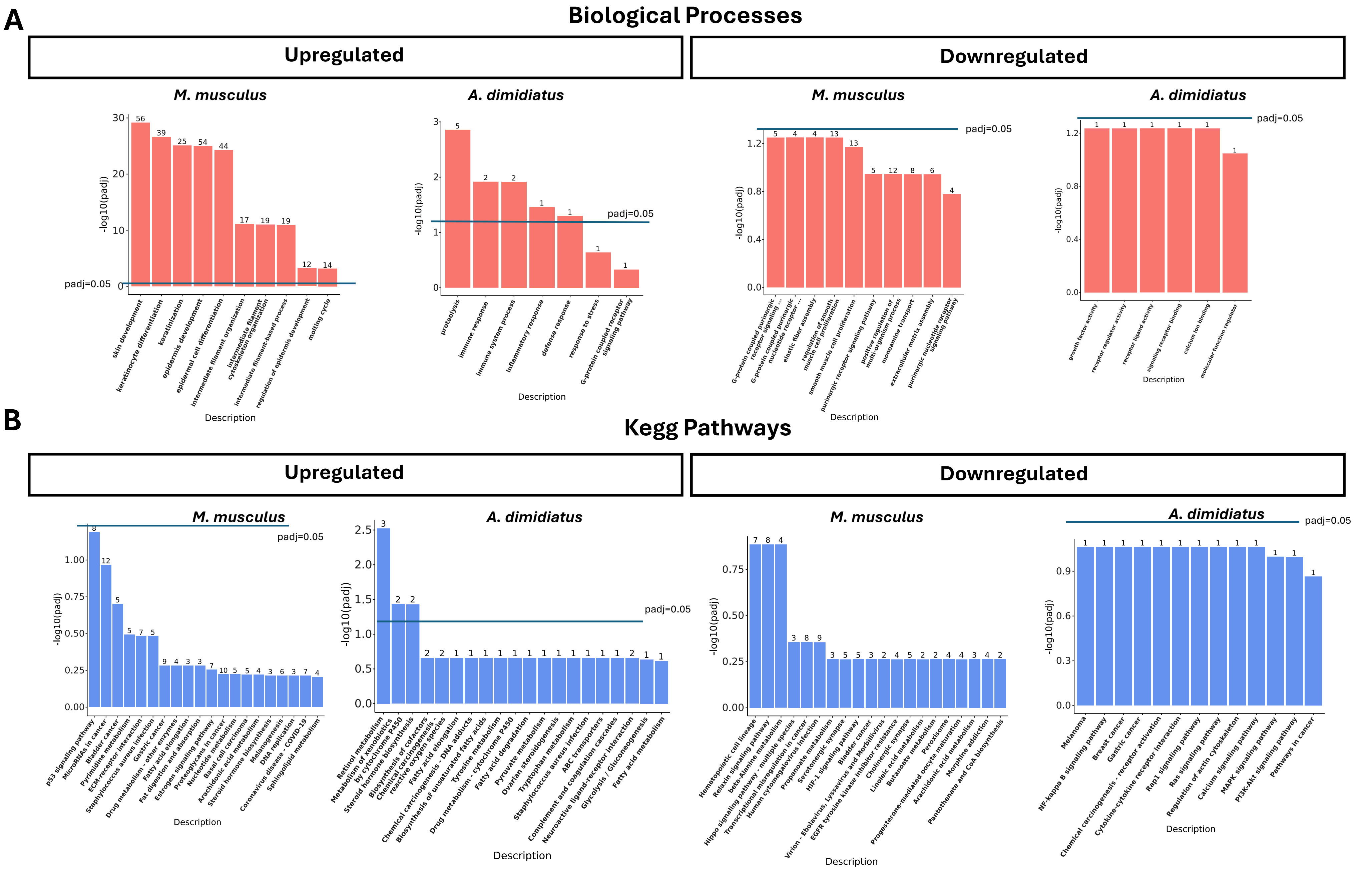

Supplementary Figure 2 - DMBA treatment upregulated several GO biological processes and Kegg pathway. (A) GO Biological processes upregulated and downregulated in *Mus musculus* and *Acomys dimidiatus*, 24h after the treatment with DMBA. **(B)** Kegg pathways upregulated and downregulated in *M. musculus* and *A dimidiatus*, 24h after the treatment with DMBA.

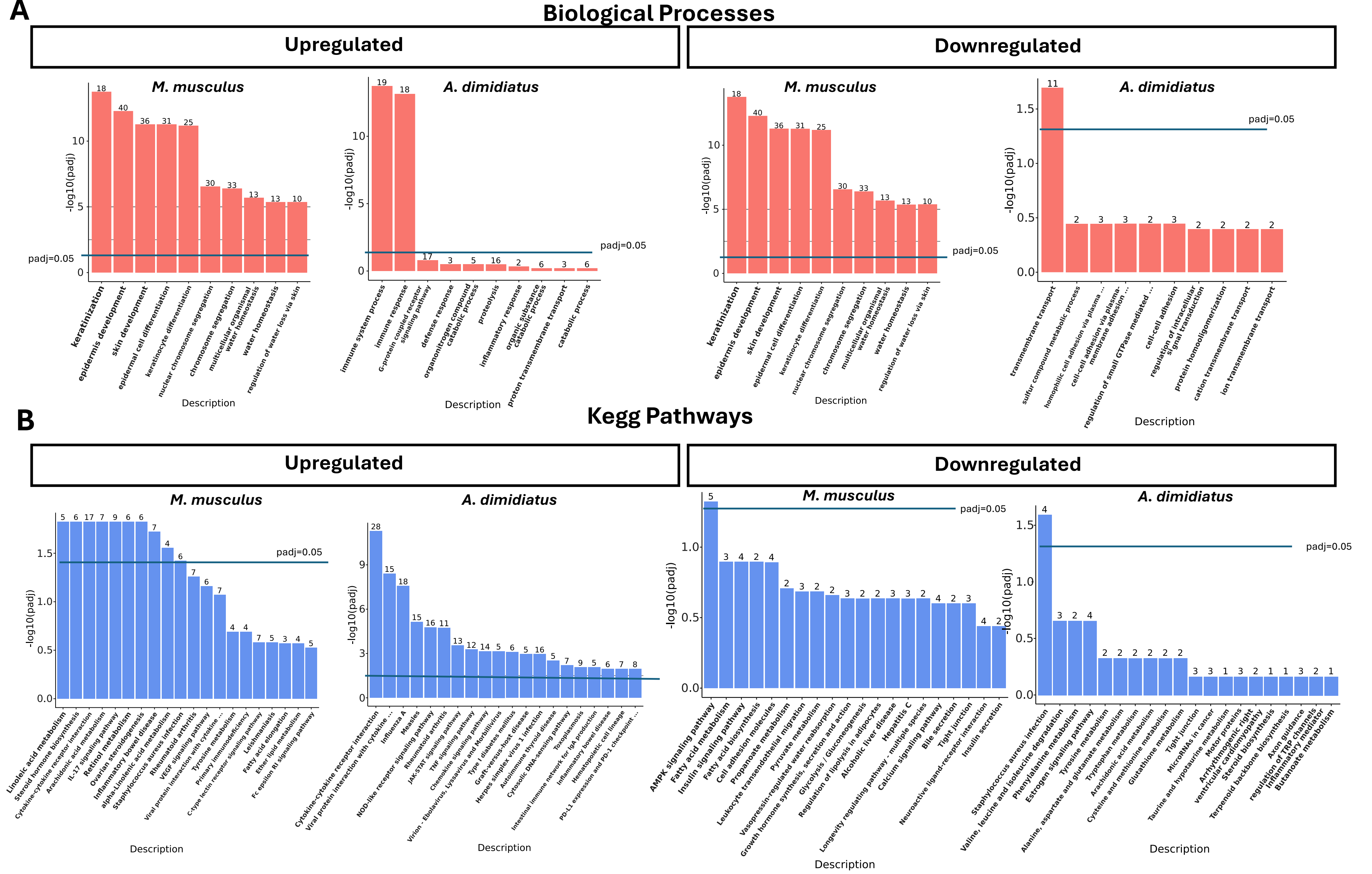

Supplementary Figure 3 – Short-term DMBA/TPA treatment upregulated several GO biological processes and Kegg pathway. (A) GO Biological processes upregulated and downregulated in *Mus musculus* and *Acomys dimidiatus*, 14 days after the treatment with DMBA. **(B)** Kegg pathways upregulated and downregulated in *M. musculus* and *A dimidiatus*, 14 days after the treatment with DMBA.

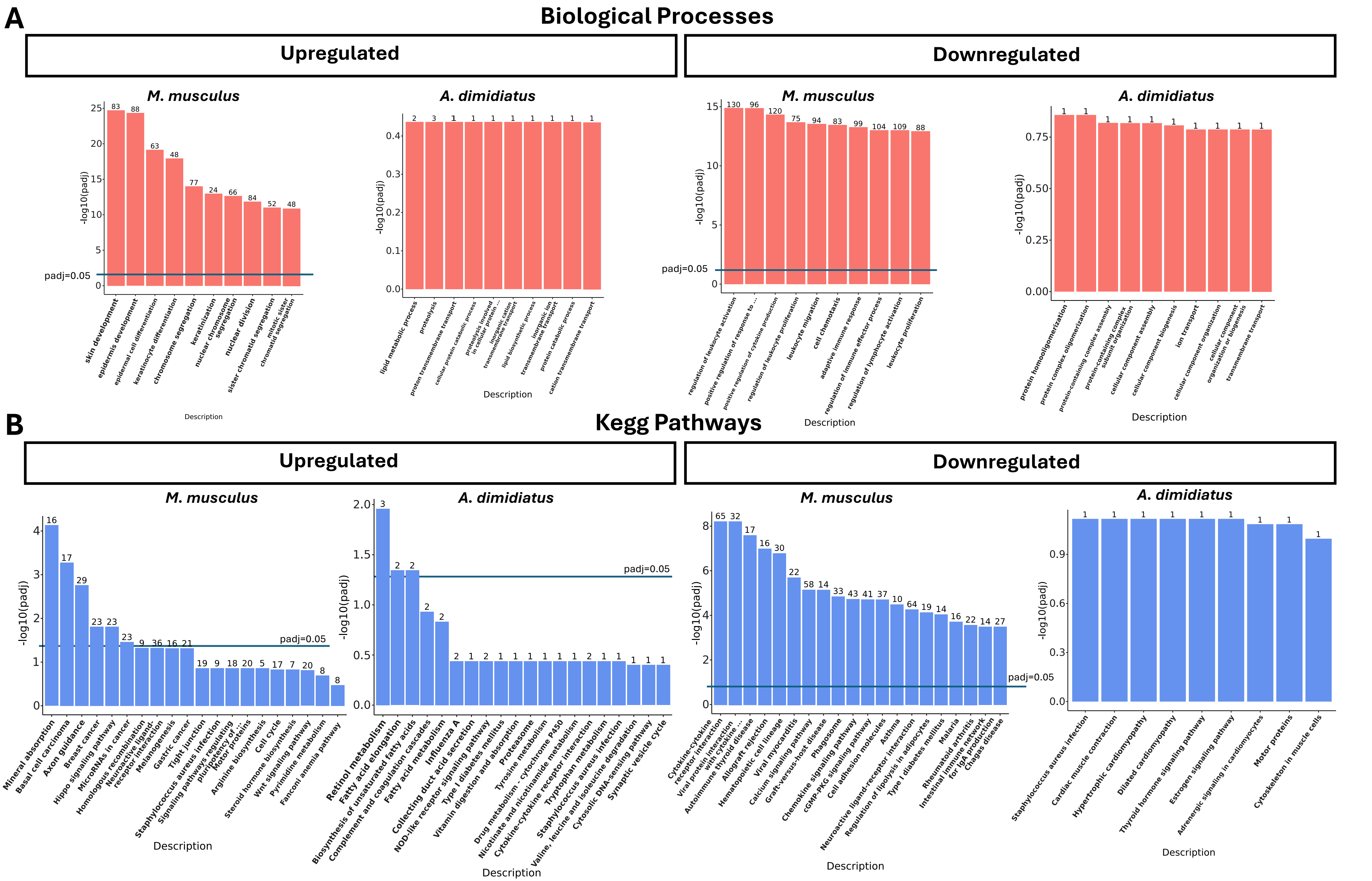

Supplementary Figure 4 – Short-term DMBA/TPA treatment during 28 days upregulated several GO biological processes and Kegg pathway in *Mus* but not in *Acomys*. (A) GO Biological processes upregulated and downregulated in *Mus musculus* and *Acomys dimidiatus*, 28 days after the treatment with DMBA. **(B)** Kegg pathways upregulated and downregulated in *M. musculus* and *A dimidiatus*, 28 days after the treatment with DMBA.
